# Downstream genes of Pax6 revealed by comprehensive transcriptome profiling in the developing rat hindbrain

**DOI:** 10.1186/1471-213X-10-6

**Published:** 2010-01-18

**Authors:** Keiko Numayama-Tsuruta, Yoko Arai, Masanori Takahashi, Makiko Sasaki-Hoshino, Nobuo Funatsu, Shun Nakamura, Noriko Osumi

**Affiliations:** 1 Division of Developmental Neuroscience, Center for Translational and Advanced Animal Research (CTAAR), Tohoku University Graduate School of Medicine, 2-1 Seiryo-machi, Aoba-ku, Sendai 980-8575, Japan; 2 Core Research for Evolutionary Science and Technology (CREST), Japan Science and Technology Agency (JST), 4-1-8 Honcho, Kawaguchi, Saitama 332-0012, Japan; 3 Max-Planck Institute of Molecular Cell Biology and Genetics, Dresden D-01307, Germany; 4 Department of Developmental and Cell Biology, School of Biological Sciences, University of California Irvine, Medical Sciences I, Irvine, CA 92697-4800, USA; 5 Department of Biotechnology and Life Science, Tokyo University of Agriculture and Technology, 2-24-16 Nakacho, Koganei, Tokyo 184-8588, Japan

## Abstract

**Background:**

The transcription factor Pax6 is essential for the development of the central nervous system and it exerts its multiple functions by regulating the expression of downstream target molecules. To screen for genes downstream of Pax6, we performed comprehensive transcriptome profiling analyses in the early hindbrain of *Pax6 *homozygous mutant and wild-type rats using microarrays.

**Results:**

Comparison of quadruplicate microarray experiments using two computational methods allowed us to identify differentially expressed genes that have relatively small fold changes or low expression levels. Gene ontology analyses of the differentially expressed molecules demonstrated that Pax6 is involved in various signal transduction pathways where it regulates the expression of many receptors, signaling molecules, transporters and transcription factors. The up- or down-regulation of these genes was further confirmed by quantitative RT-PCR. *In situ *staining of *Fabp7*, *Dbx1, Unc5h1 *and *Cyp26b1 *mRNAs showed that expression of these transcripts not only overlapped with that of *Pax6 *in the hindbrain of wild-type and *Pax6 *heterozygous mutants, but also was clearly reduced in the hindbrain of the *Pax6 *homozygous mutant. In addition, the *Pax6 *homozygous mutant hindbrain showed that *Cyp26b1 *expression was lacked in the dorsal and ventrolateral regions of rhombomeres 5 and 6, and that the size of rhombomere 5 expanded rostrocaudally.

**Conclusions:**

These results indicate that *Unc5h1 *and *Cyp26b1 *are novel candidates for target genes transactivated by Pax6. Furthermore, our results suggest the interesting possibility that Pax6 regulates anterior-posterior patterning of the hindbrain via activation of Cyp26b1, an enzyme that metabolizes retinoic acid.

## Background

Pax6 is a highly conserved transcription factor that contains two DNA-binding domains, i.e., a paired domain (PD) and a homeodomain. Pax6 has been identified as an essential regulator for the development of the central nervous system (CNS), eyes, nose, pancreas and pituitary gland, mostly through study of the phenotypes of several mouse and rat lines that possess either a spontaneous or an artificial mutation in their *Pax6 *gene. Although *Pax6 *heterozygous mutant mice/rats with a semi-dominant mutation are known as *Small eye *(*Sey*) mutants and can be bred, their homozygous mutant embryos die soon after birth and exhibit severe phenotypes such as a lack of eyes and nose as well as brain malformation. One of the rat *Small eye *strains, *rSey*^2^, has a spontaneous single-base insertion in the coding region of *Pax6 *that creates an abnormal stop codon downstream of the PD [1]. However, in Western blot analysis using an antibody against the PD of Pax6, truncated Pax6 protein was not detected in homozygous (*rSey*^2^/*rSey*^2^) embryos, although we detected truncated Pax6 protein in E10.5 *Sey/Sey *mice [2]. Therefore, *rSey*^2^/*rSey*^2 ^embryos are suitable models for analyzing the role of the Pax6 transcription factor.

In the development of the mammalian brain, Pax6 is expressed in specific spatiotemporal patterns. Pax6 is expressed in proliferating neuroepithelial cells immediately prior to the onset of neurogenesis on embryonic day 10 (E10) in rats (which corresponds to E8 in mice) and is localized to the forebrain, hindbrain and spinal cord. Pax6 regulates a variety of developmental events, including the patterning of the forebrain, hindbrain and spinal cord as well as subsequent neuronal specification, formation of neural circuits and neuronal migration [3]. Furthermore, Pax6 is involved in neurogenesis, i.e., the proliferation of neural stem/progenitor cells and production of neurons [4-6], and also in the differentiation of astrocytes [7]. The multiple functions of Pax6 are very likely exerted through the activation of its various downstream target genes.

We previously used DNA microarray analysis to demonstrate that the brain-type fatty acid binding protein (*Fabp7*) gene is markedly down-regulated in both the forebrain and the hindbrain of E12.5 *rSey*^2^/*rSey*^2 ^rats (which correspond to E10.5 mice) and is required for the maintenance of proliferating neural stem cells in the developing rat cortex [8]. Three other DNA microarray studies comparing the developing telencephalon of the *Pax6 *mutant mouse with that of the wild-type (WT) identified other Pax6 downstream genes such as *mEvf1 *[9], *Ctnnd2 *(*δ-catenin*) [10], *Starb2*, *Nfia*, *AP-2γ*, *NeuroD6*, *Ngn2*, *Tbr2*, *Bhlhb5 *and the retinoic acid (RA) signaling molecule *Rlbp1 *[11]. Recently, Pax6 target genes were identified in the developing mouse neocortex using ChIP-chip analysis in combination with transcriptome analyses, revealing Pax6-regulated transcriptional networks in the E12.5 mouse cortex [12]. Compared with these telencephalon data, less information exists on downstream genes of Pax6 in the hindbrain, although Pax6 is crucial in the development of hindbrain motor neurons, the cerebellum and the precerebellar nuclei [1,13-18].

In this study, we analyzed the transcriptome profiles of E11.5 *rSey*^2^/*rSey*^2 ^and WT rats to comprehensively identify downstream genes of Pax6 in the early stage of hindbrain development. We were able to select genes with significantly different expression using quadruplicate microarray data processed via two comparative analyses; we then confirmed the fold changes using quantitative RT-PCR. Gene ontology analyses revealed that a number of the differentially expressed genes encode signaling molecules as well as transcription factors related to development. Detailed analyses of the expression patterns of these downstream genes, including *Dbx1*, *Unc5h1 *and *Cyp26b1*, as well as several hindbrain-specific markers unexpectedly revealed anteroposterior patterning defects in the *rSey*^2^/*rSey*^2 ^hindbrain that may be related to altered expression of Cyp26b1, a retinoic acid (RA)-metabolizing enzyme.

## Results

### Comprehensive transcriptome analyses in E11.5 *rSey*^2^/*rSey*^2 ^and WT rat hindbrains

We performed the microarray experiments using GeneChip Rat Expression Set 230 (RAE230A and RAE230B), which contains about 30,000 probe sets that are expected to cover almost all genes. The two methods of comprehensive transcriptome analyses are shown in Additional file 1. Four sets of *rSey*^2^/*rSey*^2 ^and WT 'target' samples were prepared from mRNA independently extracted from 40 hindbrains of E11.5 embryos and were subjected to comparison analyses using Microarray Suite (MAS). In parallel, raw data derived from eight absolute analyses per sub-chip by MAS were exported to GeneSpring, normalized, averaged and compared. Ideally, each of the two fold-change values from both procedures should match.

**Figure 1:**
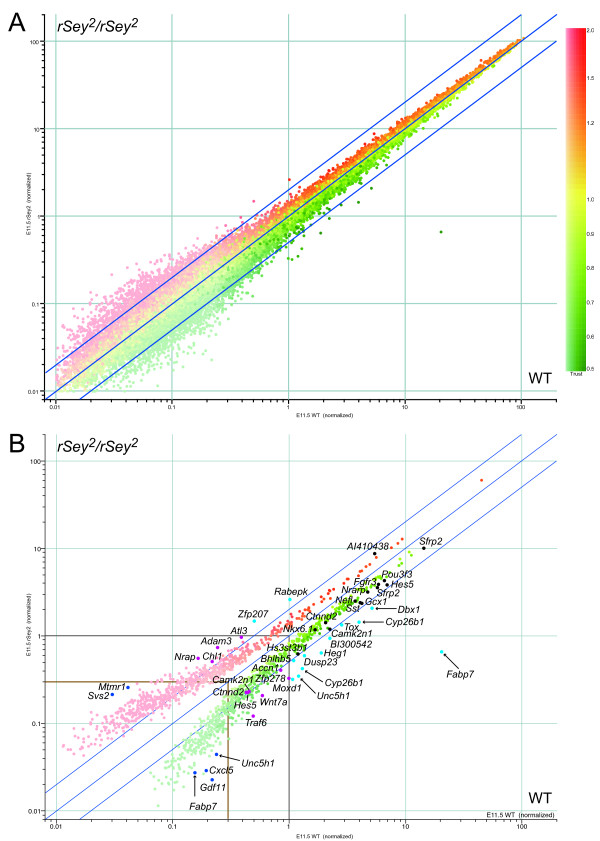
**Scatter plots of gene expression levels in the hindbrain of E11.5 *rSey*^2^/*rSey*^2 ^and WT rat embryos**. (A) Average per chip normalized signal intensities for all probe sets on GeneChip 230A and 230B are plotted using GeneSpring software. The dots are colored according to their per gene normalized values for *rSey*^2^/*rSey*^2 ^(vertical) compared to WT (horizontal): high (red), equal (yellow) and low (green). The reliability of the data is indicated by a dark-light gradient; genes with higher confidence are deeper in color. The three blue lines correspond to 2-fold up-regulation (+2), no change (± 1) and 2-fold down-regulation (-2) for *rSey*^2^/*rSey*^2 ^compared to WT. (B) The 1,421 differentially expressed genes selected by *t*-test using the cross-gene error model (*p *< 0.05). Gray and brown lines indicate the normalized signal as 1 and 0.3, respectively. Cyan, black, magenta and blue dots with gene names correspond to the probe sets that are listed in Additional file 4 and displayed in Figure 3. The colors of the dots reflect expression levels and fold change values: cyan, high expression (normalized signal > 1) with more than a 2-fold change; black, high expression with less than a 2-fold change; magenta, medium expression (normalized signal 0.3 to 1); and blue, low expression (normalized signal < 0.3) with more than a 5-fold change. The seven probe sets indicated with arrows correspond to four of the most down-regulated genes, which are shown *in situ *in Figures 4 and 5.

All genes on the arrays were plotted on a logarithmic scale in Figure 1A according to their normalized signal intensities in the hindbrain of E11.5 WT (horizontal axis) and *rSey*^2^/*rSey*^2 ^(vertical axis) rat embryos using GeneSpring. Up- and down-regulated genes are indicated in red and green, respectively. The use of four replicates greatly improved erratic patterns in areas of low signal intensity, in which data were widely spread in each of these analyses (data not shown). We did not exclude genes with the MAS-absent (A) flag from subsequent analyses because some of the genes judged to be absent (A) in all samples, such as *Ncam1*, are known to have a low expression level and/or a restricted expression pattern in the E11.5 hindbrain. For accurate selection of differentially expressed genes, we performed *t*-tests using the cross-gene error model for all genes instead of using the fold change threshold. The 1,421 selected genes with *p*-values less than 0.05 consisted of 737 up-regulated and 684 down-regulated genes (Figure 1B; Additional file 2). The up- and down-regulated genes that had relatively smaller (< 2-fold) changes (within the blue lines in Figure 1A, B) passed the *t*-test at middle and higher signal intensities (> 0.3 in both WT and *rSey*^2^/*rSey*^2^), but were discarded at the lower signal intensity (< 0.3 in either WT or *rSey*^2^/*rSey*^2^). Thus, this strategy enabled us to identify up- and down-regulated genes regardless of expression levels and fold changes.

### Gene ontology analyses of Pax6 downstream molecules

**Figure 2:**
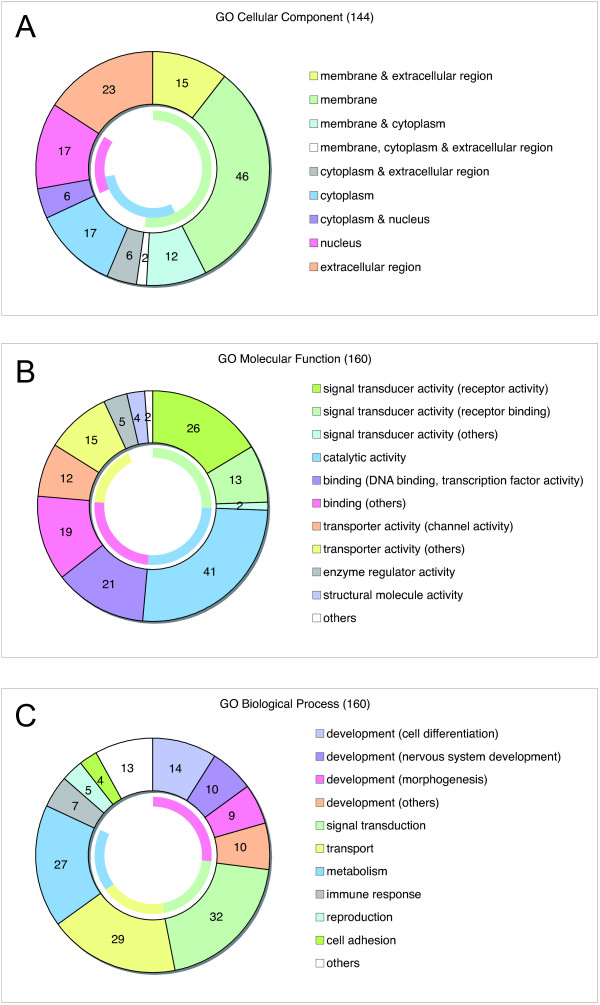
**Gene ontology (GO) analyses of the genes that were differentially expressed between *rSey*^2^/*rSey*^2 ^and WT rat hindbrains at E11.5**. Annotated genes were selected by *t*-test using the cross-gene error model (*p *< 0.05). The genes are categorized by the following GO terms: cellular component (144, A), molecular function (160, B) and biological process (160, C), and are displayed in doughnut charts. Internal color arcs indicate the total rates for each category: (A) membrane (green, 52.1%), cytoplasm (blue, 29.9%) and nucleus (red, 16.0%); (B) signal transducer (green, 25.6%), catalytic (blue, 25.6%), binding (red, 25.0%) and transporter (yellow, 16.9%) activities; and (C) development (red, 26.9%), signal transduction (green, 20.0%), transport (yellow, 18.1%), and metabolism (blue, 16.9%). The details of the annotated genes and GO terms are shown in Additional file 3.

To categorize the differentially expressed genes, we performed gene ontology (GO) analyses. GO annotations were collected for the 1,421 selected genes, and 144, 160 and 160 genes were assigned one or more GO terms: cellular component (CC), molecular function (MF), and biological process (BP), respectively (see Additional file 3). Downstream molecules corresponding to most of the EST probe sets were neither annotated nor included in the analyses. Because the GO terms provided by the GO consortium are based on a hierarchical clustering, we chose the most suitable GO term for each gene in order to simply sort them into the GO categories shown in Figure 2. Categorization by the GO term CC revealed that 75/144 (52%) genes encoded membrane components (green in Figure 2A). Regarding the MF term, 41/160 (26%) and 12/160 (7.5%) genes coded for molecules related to signal transducer (green in Figure 2B) and channel (orange in Figure 2B) activities, respectively. Genes (26/32, 81%) sorted into the "signal transduction" category of the BP term (green in Figure 2C) overlapped with the genes categorized as signal transducer and channel activities of the MF term. These results suggest that many downstream molecules of Pax6 that are located in or near the membrane function as signal transducers (e.g., *Htr4*, *Gabrr2*, *Gna15 *and *Fgf9*) that regulate various signaling cascades. Similarly, 23/144 (16%) of the gene products were found to localize in the nucleus (red in the CC, Figure 2A), and 21/160 (13%) genes encoded transcription factors or DNA binding molecules (purple in the MF, Figure 2B). As expected, 15/21 (71%) of these transcription factors or DNA binding molecules were classified in the "development" category of the BP term (e.g., *Nkx6-1*, *Ascl1*, *Neurog1 *and *Klf5*, red in Figure 2C). Thus, the GO analyses demonstrated that Pax6 is involved in various signaling pathways where it regulates the expression of many receptors, receptor binding molecules, transporters and transcription factors.

### Confirmation of differential expression of downstream genes

Next, we used quantitative RT-PCR (Q-PCR) to confirm the expression levels of the differentially expressed genes identified in the microarray comparison analyses; Q-PCR was performed using the primers listed in Additional file 4. The genes selected for Q-PCR analysis are indicated by cyan, black, magenta and blue dots in Figure 1B.

**Figure 3:**
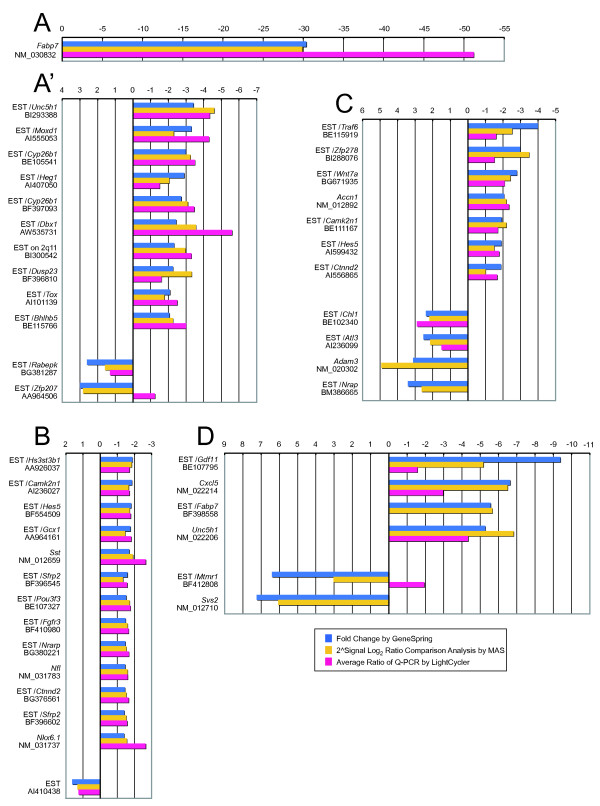
**Comparison of fold changes of up- or down-regulated genes in the E11.5 *rSey*^2^/*rSey*^2 ^rat hindbrain using three analysis methods**. The fold changes of the genes selected (*p *< 0.05) from the comparative analyses of the microarray data by GeneSpring (blue bar) and Microarray Suite (yellow bar) (shown in Additional file 1) are indicated with their real-time Q-PCR gene expression ratios (pink bar). The Q-PCR experiments were performed with LightCycler using the primers listed in Additional file 4. The genes are listed in the order of their fold change values calculated by GeneSpring. (A, A') Thirteen highly expressed genes showing high normalized signal intensity (> 1.0) in either or both conditions and high fold change value (> 2.0) in GeneSpring analyses. Since the down-regulation of *Fabp7 *is dramatic, the data are shown separately in (A). (B) Thirteen down-regulated genes and one up-regulated gene selected from the highly expressed genes with a fold change less than 2.0 in GeneSpring analyses. (C) Seven down-regulated genes and four up-regulated genes selected from genes showing medium normalized signal intensity (0.3-1.0) in both conditions. (D) Four down-regulated genes and two up-regulated genes with a high fold change (> 5.0), but a low expression level (normalized signal < 0.3). Groups of genes categorized in (A, A'), (B), (C) and (D) correspond to cyan, black, magenta and blue dots, respectively, in Figure 1B.

Among the highly expressed genes (normalized intensity of more than 1.0), all of the genes with two-fold or greater down-regulation (cyan dots in Figure 1B) showed a similar down-regulation in Q-PCR (Figure 3A, A'). The *Fabp7 *gene, which we have previously reported to be down-regulated in the E12.5 *rSey*^2^/*rSey*^2 ^rat embryo [8], showed more than 50-fold down-regulation in the hindbrain at E11.5 (Figure 3A). Another probe set for EST/*Fabp7*, located in the intronic sequence of the *Fabp7 *gene, also indicated a relatively high level of down-regulation in the two comparative microarray analyses (more than 5-fold) (Figure 3D). In addition, using Q-PCR we were able to confirm an actual decrease in expression for 13 selected genes that exhibited less than 2-fold down-regulation in the microarray analyses (black dots in Figure 1B; Figure 3B). However, one (EST/*Zfp207*) of three genes that were found to be up-regulated in the microarray exhibited down-regulation in the Q-PCR analysis (Figure 3A', B).

With respect to the selected genes that had a lower normalized intensity (between 0.3 to 1.0; magenta dots in Figure 1B), the seven genes that were down-regulated in the microarray comparison were confirmed to be down-regulated by Q-PCR, while two of four up-regulated genes were not amplified well (Figure 3C). Other ESTs for *Camk2n1*, *Hes5 *and *Ctnnd2 *exhibited similar down-regulation as shown in Figure 3B, suggesting that the normalized intensity obtained from the quadruple array samples was reliable in down-regulated genes.

Of the selected genes that had an exceptionally low normalized intensity (less than 0.3; blue dots in Figure 1B), the three down-regulated genes EST/*Gdf11*, EST/*Cxcl5 *and *Unc5h1*, which showed a similar fold change to that obtained with the probe set for intronic EST/*Fabp7*, also exhibited down-regulation in the Q-PCR analyses (Figure 3D). On the other hand, of the two selected genes that were up-regulated in the microarray, one (EST/*Mtmr1*) was down-regulated on Q-PCR and the other (*Svs2*) could not be amplified (Figure 3D). The comparative Q-PCR analyses suggest that down-regulation was more accurately reflected than up-regulation in our microarray analyses.

In further analyses of Pax6 downstream molecules, we focused on four down-regulated genes, *Fabp7*, *Dbx1*, *Cyp26b1 *and *Unc5h1*, that demonstrated higher expression in the wild type, as confirmed by both microarray (arrows in Figure 1B) and Q-PCR (Figure 3A, A', D).

### Expression patterns of Pax6 and its downstream genes in E11.5 hindbrain

**Figure 4:**
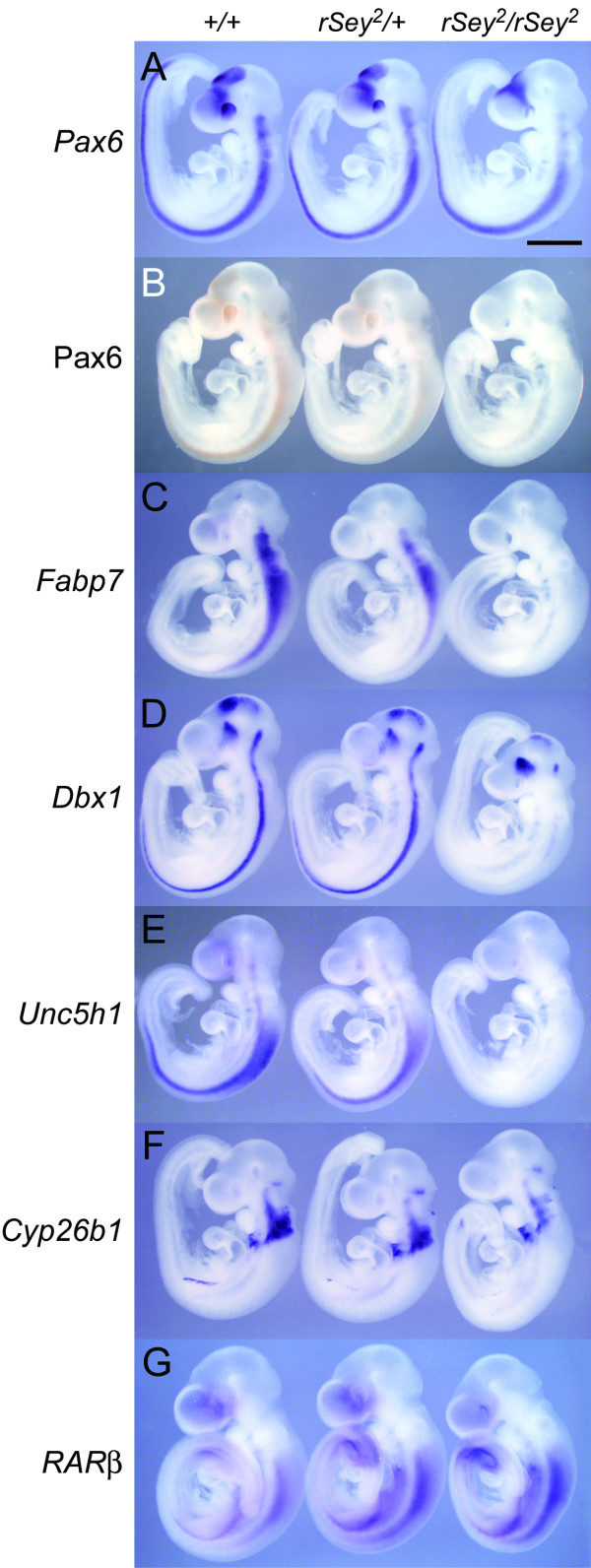
***In situ *staining of mRNA for down-regulated genes in E11.5 rat whole embryos**. (A) Whole mount *in situ *hybridization (WISH) using an antisense RNA probe for *Pax6 *[47]. Localization of *Pax6 *mRNA (blue-violet) was detected in the forebrain, hindbrain and spinal cord of all embryos of the three genotypes. (B) Immunostaining of E11.5 rat embryos with the monoclonal antibody against the PD of Pax6 [14]. The localization of the Pax6 protein (brown) is nearly identical to that of *Pax6 *mRNA in WT and *rSey*^2^/+ embryos. The expression of the Pax6 protein is reduced in the *rSey*^2^/+ embryo and is not detected in the *rSey*^2^/*rSey*^2 ^embryo. (C-F) WISH using probes for the down-regulated genes. Expression of *Fabp7 *(C) and *Unc5h1 *(E) is reduced in *rSey*^2^/+ embryos and not present in *rSey*^2^/*rSey*^2 ^embryos. While there were no significant differences found between WT and *rSey*^2^/+ embryos with respect to the expression of *Dbx1 *(D) and *Cyp26b1 *(F), the expression of these mRNAs was markedly decreased in the hindbrain (D, F) and spinal cord (D) of the *rSey*^2^/*rSey*^2 ^embryo. (G) WISH using a probe for *RARβ*. The expression of *RARβ *is up-regulated in the *rSey*^2^/+ embryo and is up-regulated to a greater extent in the *rSey*^2^/*rSey*^2 ^embryo. Scale bar: 1 mm.

Whole mount *in situ *hybridization (WISH) was performed on E11.5 embryos of three genotypes (WT, *rSey*^2^/+ and *rSey*^2^/*rSey*^2^) to examine the localization of the mRNA transcribed from four of the most down-regulated genes (i.e., *Fabp7*, *Dbx1*, *Unc5h1 *and *Cyp26b1*) as compared to *Pax6 *transcripts and protein (Figure 4). Although *Pax6 *mRNA was detected in the *rSey*^2^/*rSey*^2 ^and *rSey*^2^/+ embryos in an expression pattern similar to that of WT embryos (Figure 4A), the Pax6 protein was undetectable in the *rSey*^2^/*rSey*^2 ^embryo and seemed to be reduced in the *rSey*^2^/+ embryo as compared with WT (Figure 4B); this result was consistent with Western blot results from a previous study [2]. In the WT embryo, expression patterns of *Fabp7 *were quite similar to those of Pax6; strong signal was observed in the hindbrain and, at week one, in the dorsal telencephalon, a part of the diencephalon, as well as the spinal cord (Figure 4A, C). In the E11.5 *rSey*^2^/*rSey*^2 ^embryo, expression of *Fabp*^7 ^disappeared (Figure 4C), as previously reported for E12.5 embryos [8]. Moreover, the expression of *Fabp7 *seemed to be entirely reduced in the *rSey*^2^/+ embryo as compared with the WT embryo (Figure 4C), suggesting that the amount of Pax6 protein is critical for the induction of the *Fabp7 *gene.

*Dbx1*, which encodes a homeodomain transcription factor, is expressed in interneuron progenitor cells in the hindbrain and shows reduced expression in the E12.5 *rSey*^2^/*rSey*^2 ^hindbrain [16]. As shown in Figure 4D, *Dbx1 *expression in the *rSey*^2^/+ embryo did not differ from that of the WT embryo at E11.5. However, *Dbx1 *expression in the *rSey*^2^/*rSey*^2 ^embryo was greatly reduced, specifically in the diencephalon, hindbrain and spinal cord; these regions lack Pax6 function, as indicated by the lack of Pax6 protein in the *rSey*^2^/*rSey*^2 ^embryo (Figure 4D).

Unc5h1 is a Netrin-1 receptor that is initially expressed in neuronal progenitor cells in the ventral hindbrain [19]. *Unc5h1 *mRNA was detected in part of the forebrain as well as in the hindbrain and spinal cord in the E11.5 WT embryo; the expression domain was dorsally widened in the caudal hindbrain and anterior spinal cord (Figure 4E). *Unc5h1 *expression was reduced in the *rSey*^2^/+ embryo and was entirely absent in the *rSey*^2^/*rSey*^2 ^embryo (Figure 4E).

Cyp26b1 is an RA-catabolizing enzyme that regulates RA signaling by promoting its degradation [20]. *Cyp26b1 *showed a rhombomere-specific expression pattern in the WT hindbrain (Figure 4F), corroborating a previous report in mouse embryos [21]. *Cyp26b1 *expression in the *rSey*^2^/+ embryo did not differ from that of the WT embryo, whereas it was decreased specifically in the caudal region of the *rSey*^2^/*rSey*^2 ^hindbrain (Figure 4F).

**Figure 5:**
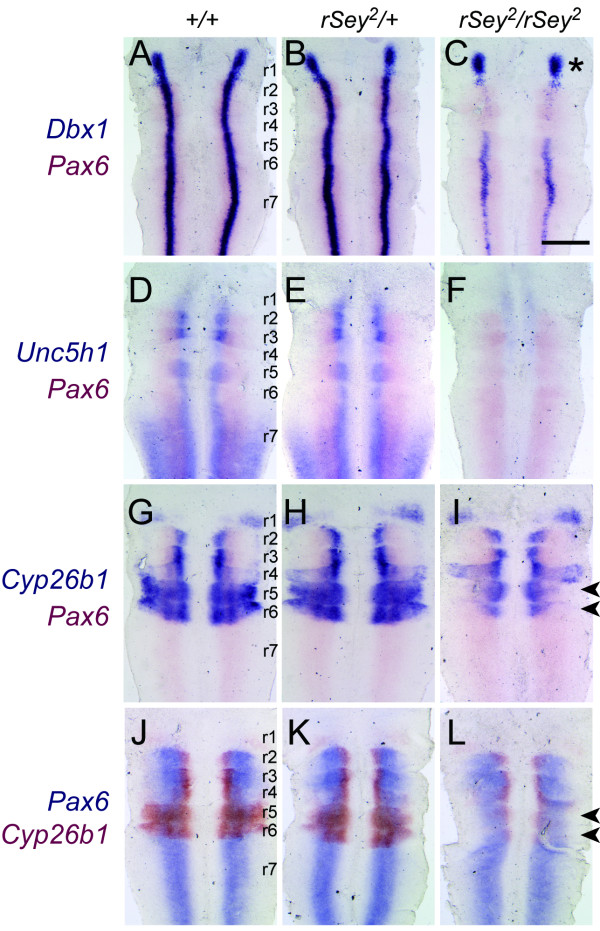
**Double *in situ *staining of mRNAs for down-regulated genes and *Pax6 *in the E11.5 rat hindbrain**. The expression sites of *Dbx1*, *Unc5h1 *and *Cyp26b1 *(blue-violet in A-I and red in J-L) were compared with that of *Pax6 *(pink in A-I and blue in J-L) in WT (A, D, G, J), *rSey*^2^/+ (B, E, H, K) and *rSey*^2^/*rSey*^2 ^(C, F, I, L) hindbrains in 'open-book' preparations. (A-C) *Dbx1 *expression is decreased throughout the interneuron progenitor domain of the *rSey*^2^/*rSey*^2 ^hindbrain with the exception of r1, where *Pax6 *is not expressed (asterisk in C). (D-F) *Unc5h1 *expression disappears in the somatic motor neuron progenitor domain and in the dorsal region of r7 in the *rSey*^2^/*rSey*^2 ^hindbrain (F). (G-L) *Cyp26b1 *expression is specifically altered in the dorsolateral region of r5 and r6 in the *rSey*^2^/*rSey*^2 ^hindbrain (arrowheads in I and L). r1 to r7 indicate the individual rhombomeres of the hindbrain. Scale bar: 500 μm.

The above results indicate that not only *Fabp7 *and *Dbx1 *but also *Unc5h1 *and *Cyp26b1 *are good candidates for Pax6 downstream target molecules in the developing CNS. To ascertain the detailed expression of these downstream genes in the E11.5 hindbrain, we prepared 'open-book' samples of this tissue (Figure 5). Double staining for *Dbx1 *and *Pax6 *demonstrated that expression of *Dbx1 *in the interneuron progenitor domain along the anterior-posterior axis overlapped with *Pax6 *in both WT and *rSey*^2^/+ hindbrains (Figure 5A, B). *Dbx1 *expression was dramatically reduced throughout the *rSey*^2^/*rSey*^2 ^hindbrain with the exception of r1 (asterisk in Figure 5C), in which *Pax6 *was not expressed. *Unc5h1 *expression disappeared in the *rSey*^2^/*rSey*^2 ^hindbrain in the ventral neuron progenitor domains and in the dorsal part of r7, where *Pax6 *was not detected at E11.5 (Figure 5F). *Cyp26b1 *expression was altered specifically in the dorsolateral region of r5 and r6 in the *rSey*^2^/*rSey*^2 ^hindbrain (indicated by arrowheads in Figure 5I, L). Thus, we consider these three genes to be very promising candidates for downstream targets of Pax6. We performed further detailed analyses focusing on *Cyp26b1 *because this gene product is involved in RA metabolism, which is an important element in the anterior-posterior patterning of the hindbrain.

### Abnormal development of posterior rhombomeres in the *rSey*^2^/*rSey*^2^

**Figure 6:**
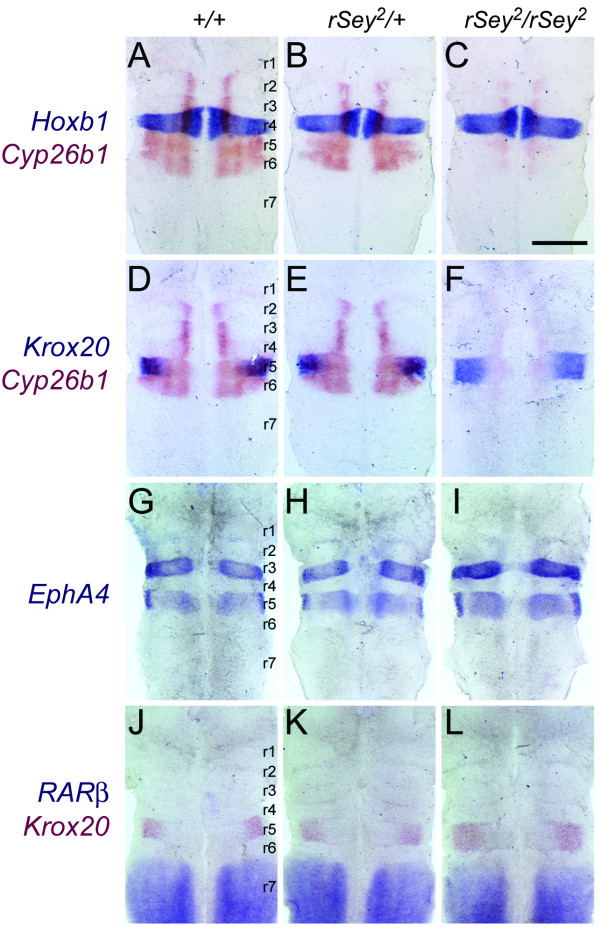
**The rhombomere 5 region is broadened in the *rSey*^2^/*rSey*^2 ^hindbrain**. (A-F) The expression sites of *Hoxb1 *and *Krox20 *(blue) were compared with that of *Cyp26b1 *(red) in WT (A, D), *rSey*^2^/+ (B, E) and *rSey*^2^/*rSey*^2 ^(C, F) hindbrains. (A-C) *Hoxb1 *expression (as a marker of r4) is unchanged in the *rSey*^2^/*rSey*^2 ^hindbrain (C). (D-F) *Krox20 *expression (as a marker of r5; cannot detect in r3 at E11.5) overlaps with *Cyp26b1 *expression in the dorsolateral domain of the WT and *rSey*^2^/+ hindbrain (D and E). The r5 region is slightly broadened in the *rSey*^2^/*rSey*^2 ^hindbrain along the anterior-posterior axis (F). (G-I) *EphA4 *expressed in both r3 and r5 of the E11.5 hindbrain. *EphA4 *expression domains (blue) are broadened only within the r5 region of the *rSey*^2^/*rSey*^2 ^hindbrain (I) as compared to the WT (G). (J-L) The expression site of *RARβ *(blue) was compared with that of *Krox20 *(red) in WT (J), *rSey*^2^/+ (K) and *rSey*^2^/*rSey*^2 ^(L) hindbrains. *Krox20 *expression is posteriorly expanded in the *rSey*^2^/*rSey*^2 ^hindbrain (L), although the anterior border of *RARβ *expression (anterior edge of r7 in L) is not altered. Scale bar: 500 μm.

To explore the rhombomere-specific alteration of *Cyp26b1 *expression, several markers were examined by double-color WISH in E11.5 WT, *rSey*^2^/+ and *rSey*^2^/*rSey*^2 ^hindbrains (Figure 6). *Hoxb1 *expression in r4 did not show any change in the three genotypes (Figure 6A-C), suggesting that r4 was intact. *Krox20 *expression was observed in r5 at this stage in the rat embryo (Figure 6D-F). In the *rSey*^2^/*rSey*^2 ^hindbrain, this *Krox20*-positive r5 region seemed to expand along the anteroposterior axis (Figure 6F; also see Figure 6J-L). A similar change in r5 morphology was also detected in the *rSey*^2^/*rSey*^2 ^hindbrain using a probe for *EphA4*, which was expressed in r3 and r5 (Figure 6G-I). r6 was found to be intact in the *rSey*^2^/*rSey*^2 ^hindbrain through the use of an *EphrinB2 *probe (data not shown).

Because the Cyp26b1 enzyme metabolizes RA, we investigated the expression of retinoic acid receptor β(*RAR*β), a gene transcriptionally induced by RA [22,23]. We expected that down-regulation of *Cyp26b1 *in the *rSey*^2^/*rSey*^2 ^hindbrain might induce up-regulation of *RAR*β in r7 and/or an anterior shift of *RAR*β to r5/6. Although we did not detect a significant increase in *RAR*β expression in our microarray analyses (Figure 1), our *in situ *analyses revealed a relatively intense signal for *RAR*β mRNA (Figure 4G) with no obvious anterior shift (Figure 6L). These results suggest that loss of Pax6 function might have resulted in slightly abnormal RA signaling, which, in turn, might cause defects in the anterior-posterior patterning of the hindbrain.

## Discussion

### Advantages of our comparative microarray analyses

We performed comprehensive transcriptional profiling analyses to search for downstream genes regulated by the Pax6 transcription factor in the E11.5 hindbrain of WT and *rSey*^2^/*rSey*^2 ^embryos using commercial high-density oligonucleotide arrays. After collecting four pools of 40 hindbrains dissected from E11.5 rat embryos of each genotype, we prepared four sets of cDNA pools for comparative microarray analyses. The advantages of our "pooled method" are: (1) the minimization of individual differences in embryonic stage, size and gene expression level and (2) the isolation of a sufficient amount of RNA for microarray analyses without unnatural amplification, even from small embryos. We believe that this "pooled method," although laborious, significantly improves the acquisition of reliable microarray data. Furthermore, comparison analyses of quadruplicate samples using two computational methods allowed us to identify differentially expressed genes that have relatively small fold changes, such as *Camk2n1*, *Hes5 *and *Ctnnd2 *(Figure 3B, C), or low expression levels, such as *Gdf11 *and *Cxcl5 *(Figure 3D). Confidence, as shown by color strength in Figure 1, indicates that the selected genes with a relatively lower fold change were also considered reliably identified in our microarray analyses.

We have succeeded in minimizing false positive/negative candidates for differentially expressed genes, especially with respect to down-regulated genes. All down-regulated genes showed a similar trend in two comparative microarray analyses and Q-PCR (Figure 3). Only two of 40 tested genes exhibited expression levels in Q-PCR that were inconsistent with their signal intensities from the microarray analyses. We could not perform Q-PCR for the three genes whose signal intensities were less than 1.0. These five genes were up-regulated in the microarray analyses. One reason for this difference is that we designed primers for Q-PCR based on full-length mRNA sequences or Ensembl/N-SCAN/Genscan prediction and not on the same EST sequences used for the probe sets. Transcription of EST domains and full-length mRNA as well as amputation/digestion of mRNA after transcription might be differentially regulated. The other reason for this difference might be the developmental stage of our samples. We used E11.5 rat hindbrains (which correspond to E9.5 in the mouse) in order to look for downstream genes that are directly regulated by Pax6. This stage occurs only 24 h after the onset of Pax6 expression. Therefore, down-regulated expression in *rSey*^2^/*rSey*^2 ^is reproducible whereas up-regulated expression, which may be indirect, is not. Reflecting on previous results from the microarray analyses of *Sey/Sey *mice, Duparc et al. [10] analyzed E9.5 *Pax6*^*LacZ/LacZ *^mouse forebrain and showed far fewer up-regulated genes as compared with down-regulated genes in a scatter plot. Other profiling analyses, in which embryos at later stages were used, have reported a larger number of up-regulated genes in *Sey/Sey *mouse telencephalon [11,12]. This finding might be attributable to the indirect regulation of an increasing number of genes by signal pathway molecules and/or transcription factors that are downstream of Pax6 at later stages of development, after the initiation of Pax6 expression. In our analyses using E11.5 *rSey*^2^/*rSey*^2 ^hindbrain, we obtained a slightly larger number of up-regulated genes (737) as compared with down-regulated genes (684), although the number of up-regulated genes with high signal intensities (68, 9.2%) was smaller than that of down-regulated genes (194, 28%) (Figure 1). This result also implies the delicacy of the molecular networks that lie downstream of Pax6 because the onset of Pax6 expression in the hindbrain occurs slightly earlier than in the forebrain.

During the course of our study, we identified multiple probe sets on the RAE230 array that recognized different sequences of the same genes and exhibited widely different signal intensities; these included the probe sets for *Fabp7*, *Unc5h1*, *Cyp26b1*, *Camk2n1*, *Hes5 *and *Ctnnd2 *(Figure 1). The variability in signal intensity among multiple probe sets may be due to (1) splicing variants, (2) different gene names and (3) ESTs that had not been integrated into the corresponding mRNA within the rat UniGene database, which was used for designing the probe sets. We used poly(A)^+ ^RNA, not total RNA, as a template, which might have resulted in a discrepancy in the signal intensities of different probes that were designed to recognize 3' or 5' sites of relatively long genes.

### Downstream factors of Pax6 clarified by GO analyses

We performed GO analyses to elucidate the overall features of the molecules downstream of Pax6. The GO analyses shown in Figure 2 revealed that various signaling molecules were located downstream of Pax6. The large quantity of information from the GO analyses enabled us to search for interesting features of the downstream factors of Pax6. For example, we found transcriptional changes in *Sst*, *Ceacam1*, *Cspg3*, *Ascl1*, *Unc5h1*, *Nrg1 *and *Thy1*, which are categorized in the "cell migration" group of the GO BP term (see Additional file 3). Because Pax6 was reported to regulate granule cell polarization and migration in the developing cerebellum [15], some of these factors might be involved in the molecular mechanisms of these events.

Gohlke et al. reported GO analyses of transcriptional profiling using mice deficient in or overexpressing *Ngn2 *and *Mash1*, key transcriptional factors that promote neurogenesis [24]. Genes were selected that were up-regulated in telencephalon tissues transfected with Ngn2 or Mash1 (i.e., samples 18 h after electroporation at E10.5) and down-regulated in telencephalon tissues of *Ngn2 *and/or *Mash1 *knockout mice at E13.5. Among the 131 and 35 genes that had GO annotations and were considered to be regulated by Ngn2 and Mash1, respectively, 32% and 37% of the genes, respectively, were categorized with the GO term "signal transduction/receptor", and 14% and 20% of the genes, respectively, were tagged with "regulation of transcription". These results agree with our list of candidate genes that might be regulated by Pax6 in the E11.5 rat hindbrain; such agreement is quite reasonable considering that Ngn2 is reported to be directly induced by Pax6 and to repress Mash1 [25,26].

### Downstream genes previously reported in *Pax6 *mutants

We previously reported that the development of cranial motor neurons in the *rSey*^2^/*rSey*^2 ^hindbrain is abnormal; two somatic motor nerve types--the abducent (VI) and hypoglossal (XII) motor neurons--are missing when Pax6 function is lost [1] owing to the abnormal formation of progenitor domains for these ventral motor neurons along the dorsoventral axis [16]. We also found that *cadherin7/cadherin20*-expressing nXII motor nuclei are absent in E18.5 *rSey*^2^/*rSey*^2 ^rats [27]. The expression levels of genes that encode progenitor domain-specific homeodomain proteins and the secreted molecule Wnt7b are altered in the ventricular zone of the E12.5 mutant hindbrain [1,16], although the identity of the other molecules involved in the downstream network of Pax6 that affects hindbrain development remains unclear.

In this microarray study, the down-regulation of *Fabp7 *and *Dbx1*, which has been previously described [8,16], was confirmed. Expression of *Fabp7 *in the hindbrain showed a greater reduction at E11.5 than at E12.5, suggesting that regulation of *Fabp7 *at the earlier stage is more dependent on Pax6 function. A previous paper reported a loss of expression of Ngn2 in the somatic motor neuron progenitor domain in the E12.5 *rSey*^2^/*rSey*^2 ^hindbrain [28]. In our analyses using E11.5 hindbrains, we could not find a significant difference in *Ngn2 *expression (*p *> 0.05; 1.4-fold by GeneSpring, 1.2-fold by MAS and 1.6-fold by Q-PCR). We also compared *Ngn2 *expression in the E11.5 hindbrain via WISH, but there was no remarkable difference between WT and *rSey*^2^/*rSey*^2 ^(Takahashi and Osumi, unpublished data). These findings suggest a temporally distinct regulation of *Ngn2 *by Pax6.

Hes family members (reviewed in [29]) and Bhlhb5 are other important bHLH transcription factors related to neurogenesis. In our microarray analyses, probe sets for *Hes5 *suggested 1.8- and 1.9-fold down-regulation (*p *< 0.05, Figure 3B, C), although *Hes1 *and *Hes3 *expression levels did not differ between WT and *rSey*^2^/*rSey*^2^. *Bhlhb5 *expression was also significantly down-regulated in the *rSey*^2^/*rSey*^2 ^hindbrain (2.1-fold, *p *< 0.05, Figure 3A'), similar to previous results of transcriptome analyses of the *Sey/Sey *mouse telencephalon [11]. Bhlhb5 was reported to be expressed in the mouse cortical primordium and to control the subtype specificity of projection neurons [30]. Furthermore, *Ngn2*, a downstream gene of Pax6, was shown to be upstream of *Bhlhb5 *[31,32]. Meanwhile, in humans, BHLHB5 is expressed in the cerebellum and can repress *Pax6 *expression [33]. Therefore, it is presumed that Pax6 regulates the proliferation and differentiation of neural stem/progenitor cells through the organization of bHLH transcription factors in a nested manner.

Although we could not detect down-regulation of *Wnt7b *in the E11.5 hindbrain as found at E12.5 [1], another member of the Wnt family, *Wnt7a*, showed significant down-regulation (Figure 3C) as reported in the mouse forebrain [11]. Therefore, the regulation of downstream genes by Pax6 seems to be quite context-dependent; Pax6 may differentially regulate the expression of target molecules according to stage and tissue.

### Novel downstream genes of Pax6 and their relationship to the anteroposterior patterning of the hindbrain

In the present study, we identified novel candidates for Pax6 downstream molecules. First, *Unc5h1 *expression was abolished in the *rSey*^2^/*rSey*^2 ^hindbrain in the ventral expression domain of *Pax6 *within r2 to r7 (Figure 4A, E and 5F). Furthermore, *Unc5h1 *expression was detected in the dorsal part of r7 and in the spinal cord (Figure 4E and 5D, E), where *Pax6 *was not detected at E11.5 (Figure 4A and 5F); however, Pax6 was expressed at an earlier stage in WT and *rSey*^2^/+ (data not shown). In the hindbrain of *rSey*^2^/*rSey*^2^, almost no expression of *Unc5h1 *was revealed by WISH (Figures 4E and 5F). From these results, we suspect that expression of *Unc5h1 *is regulated by Pax6 prior to E11.5. In the cerebellum of the *Pax6 *homozygous mutant mouse, *Unc5h3 *expression is completely absent in the external granule cell layer [14]. These findings may suggest a common regulation of expression of duplicated *Unc5 *family genes by Pax6.

Another Pax6 downstream candidate is Cyp26b1, an RA catabolic enzyme. A lack of Cyp26b1 results in the dispersion of the RA signal in the developing limb [34]. RA is thought to act as a diffusible morphogen that patterns the hindbrain along the anteroposterior axis, and degradation of RA by Cyp26s is a major determinant of spatiotemporally specific RA signaling (reviewed in [35]). RA also alters the migration patterns of cranial neural crest cells in the rat embryo [36]. We found that *Cyp26b1 *expression was specifically abolished in r5 and r6 in the *rSey*^2^/*rSey*^2 ^hindbrain (Figure 5I, L). These two rhombomeres express the *kreisler *gene, which encodes the MafB transcription factor, a protein with a critical role in caudal hindbrain patterning [37,38]. Maf partners with Pax6 for synergistic transcriptional activation of several *crystallin *and *glucagon *genes [39,40]. Therefore, the expression of *Cyp26b1 *in r5 and r6 is assumed to be regulated by Pax6 together with MafB.

Although knockout mice lacking Cyp26b1 function show no major defects in the hindbrain [34,41], two reports describe the contribution of Cyp26b1 to hindbrain development in zebrafish; Hernandez et al. found that injection of a *cyp26b1 *antisense morpholino oligonucleotide (MO) into *cyp26a1 *mutants caused expansion of r4 and an anterior shift of the r6/7 boundary [42]. Additionally, Reijntjes et al. showed, by preventing the splicing of the *cyp26b1 *transcript in WT embryos using another *cyp26b1 *MO, a reduced rhombomere size and defects in craniofacial structures at later stages [43]. In detailed analyses of rhombomeres using a *Krox20 *probe, we observed that the r5 region was broadened anteroposteriorly to some extent (Figure 6F, L). This result represents a novel finding that anterior-posterior patterning of the hindbrain is altered in the *Pax6 *mutant. Consistent with this result, we found a slight up-regulation of *Krox20 *(1.63- and 1.31-fold change with two probe sets, but it should be noted that *p *> 0.05).

Recently, the *Hoxd4 *gene was reported to be a direct target of *Pax6. Hoxd4 *expression in r7 and the spinal cord is reduced to some extent in the *Pax6 *mutant mouse embryo, and double knock-down of *pax6a *and *pax6b *with MOs resulted in malformed rhombomere boundaries and an anteriorized *hoxd4a *expression border in the zebrafish embryo [44]. In our microarray analyses, *Hoxd4 *showed slight down-regulation with statistical significance (1.37-fold; *p *< 0.05), although its expression level was quite high, even in the *rSey*^2^/*rSey*^2 ^hindbrain (normalized intensity 3.332 vs. 4.581 in WT). Therefore, we consider that a loss of Pax6 function affects the anteroposterior patterning of r5 by *Cyp26b1 *down-regulation rather than by regulation of *Hoxd4 *gene expression.

The anteroposterior defect that we observed in the *Pax6 *mutant hindbrain was very mild as compared with that of phenotypes induced by excess RA (reviewed in [45,46]). It would be interesting to determine how mildly elevated levels of RA signaling affect rhombomere formation and subsequent neural function.

## Conclusions

We identified *Unc5h1 *and *Cyp26b1 *as novel downstream genes of Pax6 in the early development of the hindbrain. In addition, the broadening of the r5 region in *Pax6 *mutant embryos raises the possibility that Pax6 regulates the anterior-posterior patterning of the hindbrain via activation of the RA-metabolizing enzyme Cyp26b1.

## Methods

### Animals

The procedures for preparing rat *Small eye *(*rSey*^2^) mutant and normal Sprague-Dawley (WT) embryos and for genotyping by mutation-specific PCR with genomic DNA extracted from each yolk sac have been described previously [2]. All experimental procedures described in this study were approved by the Committee for Animal Experimentation of the Tohoku University Graduate School of Medicine.

### Microarray analysis

Microarray analyses were performed using the GeneChip Rat Expression Arrays RAE230A and 230B (Affymetrix), which contain 31,042 probe sets that were designed from sequences in the UniGene database (NCBI, Build 99). The probe sets on RAE230A consist of the sequences from 4,699 full-length cDNAs, 700 non-ESTs and 10,467 ESTs. All 15,176 probe sets on RAE230B were derived from EST sequences.

Total RNA was prepared using TRIZOL Reagent (Invitrogen) from 40 hindbrains dissected from E11.5 *Pax6 *homozygous mutant (*rSey*^2^/*rSey*^2^) or WT rat embryos. Poly(A)^+ ^RNA was isolated with the Oligotex-dT30 <Super> mRNA Purification kit (TaKaRa). The RNA quality and concentration were confirmed by agarose gel electrophoresis and NanoDrop spectrometry (NanoDrop Technologies). The following method for the preparation of target cRNAs is similar to that in our previous report [8]. mRNA (2 μg) and a T7-linked oligo(dT) primer were used to generate first-strand cDNA with the SuperScript Choice System (Invitrogen). After second-strand synthesis, double-stranded cDNAs were purified via phenol/chloroform extraction and ethanol precipitation. *In vitro *transcription of the cDNA pools was performed using a BioArray HighYield RNA transcript labeling kit (Enzo) in the presence of biotinylated UTP and CTP, resulting in approximately 100-fold amplification of biotin-labeled cRNAs. The cRNAs were purified using RNeasy Mini Spin Columns (QIAGEN) and fragmented at 94°C for 35 min in fragmentation buffer (30 mM magnesium acetate, 100 mM potassium acetate and 40 mM Tris-acetate, pH 8.1). The resultant target cRNA fragments (20 μg) were mixed with spike controls, divided into two sub-chips (RAE230A and RAE230B Arrays), and hybridized with the oligonucleotide probes on the GeneChip at 45°C for 16 h according to the manufacturer's recommendations. After washing and streptavidin-phycoerythrin staining steps, fluorescence signals were enhanced with a biotinylated anti-streptavidin antibody using the Affymetrix fluidics station (EukGE-WS2 protocol) and detected with the Affymetrix GeneChip Scanner. Array images were assessed by eye to confirm scanner alignment and the absence of significant bubbles or scratches on the chip surface. Experiments using 40 hindbrains were repeated four times in each genotype, i.e., four pools of 40 hindbrains per genotype were used for preparing four 'target' cRNA pools per genotype, and each of the cRNA pools was hybridized to one RAE230 array.

Using Microarray Suite (MAS) 5 (Affymetrix), we performed absolute analyses for each chip to obtain raw signal intensity data as well as present (P), absent (A) and marginal (M) flags. In the MAS expression reports, 3'/5' ratios for *GAPDH *and β-*actin *were confirmed to be within acceptable limits (1.69 ± 0.69 and 1.73 ± 1.01), and *BioB *spike controls were found to be present on all of the chips, with *BioC*, *BioDN *and *CreX *also present in increasing intensity. In addition, four independent sets of comparison analyses were performed using MAS to obtain four signal log_2 _ratios of *rSey*^2^/*rSey*^2 ^to WT per gene. n was calculated as the average of the four signal log_2 _ratios, and 2^n ^corresponds to the fold change value of the gene.

### Data analysis by GeneSpring software

In further analyses, all raw signal data were exported from MAS .CHP files and imported to GeneSpring 7 software (Agilent). The raw data were transformed to set measurements of less than 0.01 to 0.01 in order to display all of the data on a logarithmic scale. In order to fit the gene expression levels from the 230A and 230B arrays, data were normalized against positive control genes and genes common to both 230A and 230B. They were then used in the following expression data analyses. The "fold change" value is the ratio of the average normalized signal intensity of *rSey*^2^/*rSey*^2 ^to that of WT for up-regulated genes (*rSey*^2^/*rSey*^2 ^> WT). In the case of down-regulated genes (*rSey*^2^/*rSey*^2 ^< WT), the fold change is the negative inverse ratio. Comparative analyses were performed using two confidence filters. First, the data for all genes were normalized per gene for the WT samples. Genes (737) with a *t*-test *p*-value from 0 to 0.05 were selected under the condition that the cross-gene error model was active, but none of the multiple testing corrections was used. For the normalization per gene for the *rSey*^2^/*rSey*^2 ^samples, 688 genes passed similar filtering conditions. The genes from the individual filter steps were combined to generate a list of 1,421 genes. No significant change was detected for *Sox2*, *Shh *or *Pax3*, which are expressed in the E11.5 hindbrain but not expected to be regulated by Pax6. In the scatter plots, the dots are colored to reflect the expression levels of the *rSey*^2^/*rSey*^2 ^samples normalized per gene for the WT samples, with color density indicating reliability. Because the GeneChip RAE 230 gene titles that were provided by Affymetrix were sometimes incorrect, the master gene table for RAE 230 was updated using GeneSpider, which automatically submitted each gene to three databases: Entrez Gene, GenBank and UniGene. If required, gene annotation was manually submitted to multiple databases including UCSC Genome Browser, NetAffx Analysis Center, RefSeq and LocusLink using Gene Inspector. The GO terms titled "GO cellular component", "GO molecular function" and "GO biological process" were used for GO analyses and are listed in the three columns of the updated master gene table for RAE230.

### Quantitative PCR

Real-time quantitative PCR was performed using a LightCycler (Roche), FastStart DNA Master SYBR Green I (Roche) and the same double-stranded cDNA templates that were used in the microarray analyses. Primer sequences for *Fabp7 *were described previously [8]. Other primer sets were designed to amplify about 100 to 150 bp cDNA fragments (Additional file 4) and were synthesized by Nihon Gene Research Laboratories Inc. The level of expression of each mRNA was normalized against that of *GAPDH*, which was amplified as an internal standard from the same cDNA pool. To ascertain whether the correct cDNAs were amplified, the size of the PCR products was determined by agarose gel electrophoresis, and then the sequences were verified by direct sequencing of the amplified fragments using the ABI310 at the Biomedical Research Core of Tohoku University Graduate School of Medicine.

### *In situ *hybridization and immunostaining

WISH as well as double staining with digoxigenin (DIG)- and fluorescein isothiocyanate (FITC)-labeled RNA probes were performed as described previously [1,27]. To standardize expression levels, WT, *rSey*^2^/+ and *rSey*^2^/*rSey*^2 ^samples were processed simultaneously. Each cDNA template for the DIG- and FITC-labeled RNA probes was amplified by RT-PCR with the following oligonucleotide primers: *Unc5h1*, 5'-TGCCTGCACACCGCTTCTTG-3' and 5'-GTGGGTGTCGTGTAGGCAGT-3'; *Cyp26b1*, 5'-ATGCTCTTTGAGGGCTTGGAGTTG-3' and 5'-CTACACCGTAGCACTCAACATGGC-3'; *Hoxb1*, 5'-CCGGACCTTCGACTGGATG-3' and 5'-GGTCGGAGGCCTCTCCAGC-3'; *Krox20*, 5'-GAGGCCCCTTTGATCAGATG-3' and 5'-GGAGAGTAGAGGTGGTCCAG-3'. The cDNAs were inserted into the *EcoRV *site of the pBluescript II SK(-) vector (Stratagene). The mouse *EphA4 *cDNA was kindly provided by Dr. Y. Hara. The templates for rat *Pax6 *Region B [47], rat *Fabp7 *[8], rat *Dbx1 *[16] and mouse *RAR*β [48] were described previously. Hindbrains were dissected from the stained embryos, cut along the dorsal midline, placed on a glass slide in an "open-book" configuration and flattened by mounting cover slips. Whole mount immunostaining was essentially performed as described previously [36]. AD2.38, a monoclonal antibody raised against the PD of Pax6 [14], was a gift from Dr. V. van Heyningen. A horseradish peroxidase (HRP)-conjugated anti-mouse IgG antibody (CHEMICON) was used as the secondary antibody. HRP was visualized by the addition of its substrates, H_2_O_2 _and 3,3'-diaminobenzidine (DAB; Dojindo), which resulted in a brownish coloration. We confirmed the expression patterns by performing these experiments at least three times.

## Authors' contributions

KN-T designed and performed the microarray experiments, data analyses, immunostaining and WISH. YA participated in the design and execution of the microarray experiments. MT aided in the double color *in situ *hybridization experiments and approved the manuscript. MS-H performed the WISH and took pictures. NF and SN assisted with the microarray experiments. NO conceived the study and participated in its design and coordination. KN-T and NO wrote the manuscript together. All authors read and approved the final manuscript.

## Supplementary Material

Additional file 1 **Schemes for the computation of fold change by two comparison analyses**. RAE230A and RAE230B are high-density oligonucleotide array sub-chips of GeneChip Rat Expression Set 230 and contain about 30,000 probe sets designed from sequences in the UniGene database. Microarray Suite (MAS) 5 (Affymetrix) and GeneSpring 7 (Agilent) were employed as the analysis software for data processing, normalization and comparison in two sets of analyses. (A) Four sets of the *rSey*^2^/*rSey*^2 ^and WT 'target' samples were hybridized to 230A and 230B arrays and scanned. The resultant signals were used in the comparison analyses by MAS. Four independent comparison analyses per sub-chip resulted in quadruplicate signal log_2 _ratios per gene. An average of the four ratios was used to calculate the powers of two in Microsoft Excel, which corresponded to the fold change for each gene in the WT and *rSey*^2^/*rSey*^2 ^samples. (B) Raw data of the signal intensity derived from eight absolute analyses per sub-chip by MAS were exported to GeneSpring and normalized to positive control genes and genes common to both 230A and 230B arrays in order to compare the expression levels of all genes on both sub-chips straightforwardly. Four sets of normalized data were averaged individually for the WT and *rSey*^2^/*rSey*^2 ^samples. The ratio of the mean is equal to the fold change per gene. Click here for file

Additional file 2 Lists of down- (A) and up- (B) regulated genes with significant differences (*p *< 0.05). Click here for file

Additional file 3 Lists of genes with the Gene Ontology terms: GO cellular component (A), GO molecular function (B) and GO biological process (C). Click here for file

Additional file 4 Sequences of primer sets for Q-PCR analyses of down- and up- regulated genes. Click here for file
