# Supplementary material for: Downstream genes of Pax6 revealed by comprehensive transcriptome profiling in the developing rat hindbrain
Source: BMC Dev Biol. 2010 Jan 18;10:6. doi: 10.1186/1471-213X-10-6 (PMC2818624; doi:10.1186/1471-213X-10-6)
Supplement: Additional file 1 — Schemes for the computation of fold change by two comparison analyses. RAE230A and RAE230B are high-density oligonucleotide array sub-chips of GeneChip Rat Expression Set 230 and contain about 30,000 probe sets designed from sequences in the UniGene database. Microarray Suite (MAS) 5 (Affymetrix) and GeneSpring 7 (Agilent) were employed as the analysis software for data processing, normalization and comparison in two sets of analyses. (A) Four sets of the rSey2/rSey2 and WT 'target' samples were hybridized to 230A and 230B arrays and scanned. The resultant signals were used in the comparison analyses by MAS. Four independent comparison analyses per sub-chip resulted in quadruplicate signal log2 ratios per gene. An average of the four ratios was used to calculate the powers of two in Microsoft Excel, which corresponded to the fold change for each gene in the WT and rSey2/rSey2 samples. (B) Raw data of the signal intensity derived from eight absolute analyses per sub-chip by MAS were exported to GeneSpring and normalized to positive control genes and genes common to both 230A and 230B arrays in order to compare the expression levels of all genes on both sub-chips straightforwardly. Four sets of normalized data were averaged individually for the WT and rSey2/rSey2 samples. The ratio of the mean is equal to the fold change per gene. [file 1471-213X-10-6-S1.PDF]

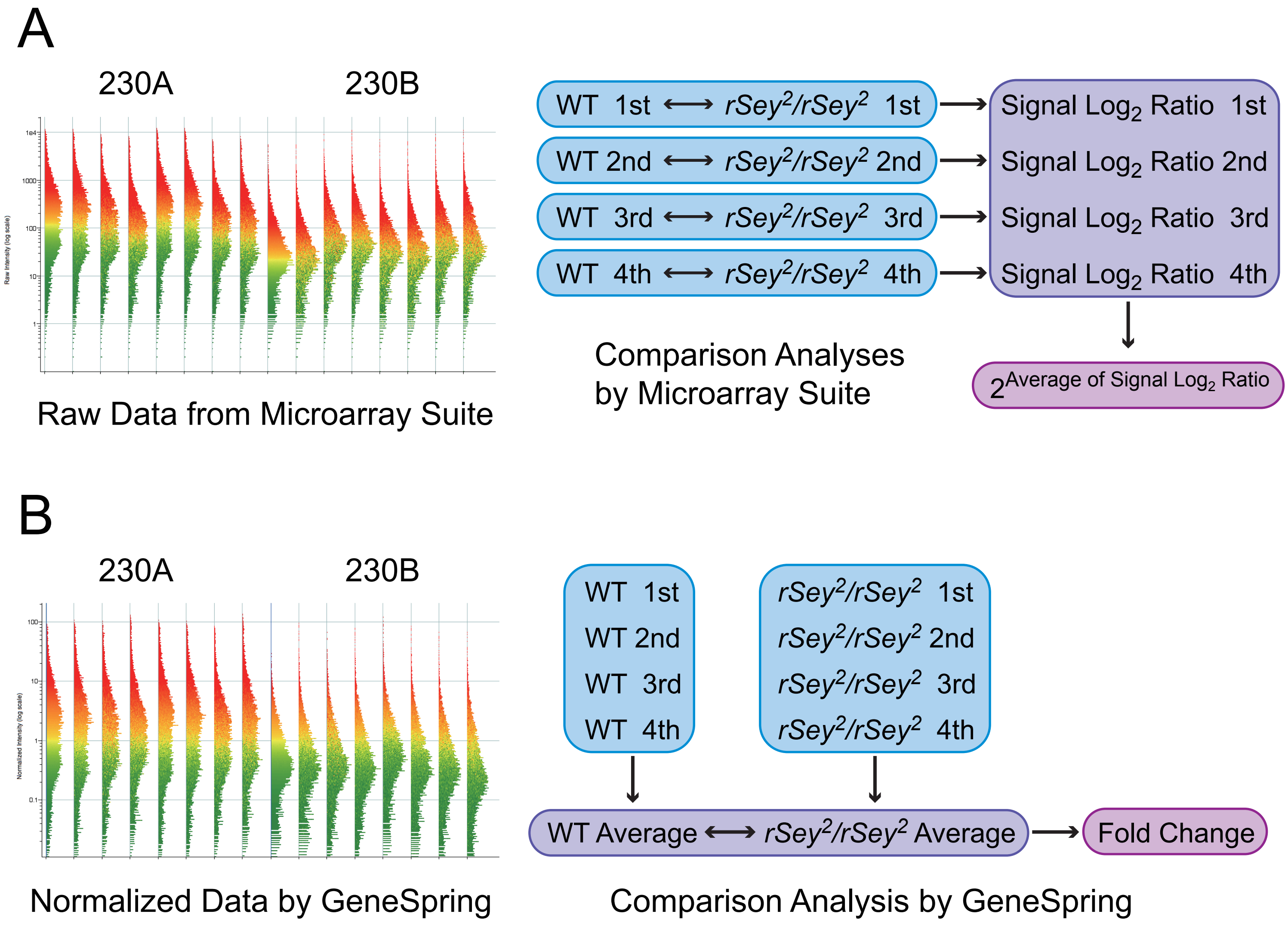

K. Numayama-Tsuruta et al. Additional file 1
